# Metaproteogenomic Profiling of Microbial Communities Colonizing Actively Venting Hydrothermal Chimneys

**DOI:** 10.3389/fmicb.2018.00680

**Published:** 2018-04-06

**Authors:** Petra Pjevac, Dimitri V. Meier, Stephanie Markert, Christian Hentschker, Thomas Schweder, Dörte Becher, Harald R. Gruber-Vodicka, Michael Richter, Wolfgang Bach, Rudolf Amann, Anke Meyerdierks

**Affiliations:** ^1^Department of Molecular Ecology, Max Planck Institute for Marine Microbiology, Bremen, Germany; ^2^Division of Microbial Ecology, Department of Microbiology and Ecosystem Science, University of Vienna, Vienna, Austria; ^3^Institute of Pharmacy, University of Greifswald, Greifswald, Germany; ^4^Institute of Microbiology, University of Greifswald, Greifswald, Germany; ^5^Department of Symbiosis, Max Planck Institute for Marine Microbiology, Bremen, Germany; ^6^Ribocon GmbH, Bremen, Germany; ^7^MARUM Center for Marine Environmental Sciences, Department of Geosciences, University of Bremen, Bremen, Germany

**Keywords:** hydrothermal vent, metagenome, metaproteome, sulfur cycling, *Epsilonproteobacteria*

## Abstract

At hydrothermal vent sites, chimneys consisting of sulfides, sulfates, and oxides are formed upon contact of reduced hydrothermal fluids with oxygenated seawater. The walls and surfaces of these chimneys are an important habitat for vent-associated microorganisms. We used community proteogenomics to investigate and compare the composition, metabolic potential and relative *in situ* protein abundance of microbial communities colonizing two actively venting hydrothermal chimneys from the Manus Basin back-arc spreading center (Papua New Guinea). We identified overlaps in the *in situ* functional profiles of both chimneys, despite differences in microbial community composition and venting regime. Carbon fixation on both chimneys seems to have been primarily mediated through the reverse tricarboxylic acid cycle and fueled by sulfur-oxidation, while the abundant metabolic potential for hydrogen oxidation and carbon fixation via the Calvin–Benson–Bassham cycle was hardly utilized. Notably, the highly diverse microbial community colonizing the analyzed black smoker chimney had a highly redundant metabolic potential. In contrast, the considerably less diverse community colonizing the diffusely venting chimney displayed a higher metabolic versatility. An increased diversity on the phylogenetic level is thus not directly linked to an increased metabolic diversity in microbial communities that colonize hydrothermal chimneys.

## Introduction

At sites of marine hydrothermal discharge, mixing of reduced venting fluids with oxygenated seawater leads to the formation of metal sulfides, sulfates, and oxides, which form hydrothermal chimneys and flanges (Schrenk et al., 2008). The development of hydrothermal deposits at venting edifices proceeds through various maturation stages and different structure types can be formed (Rickard et al., 1994; Tivey, 2004). In black smoker chimneys, characteristic of high-temperature venting (>300°C), fluids flow through crystalline conduits, resulting in the formation of sharp temperature and substrate gradients throughout the chimney walls (Tivey, 2004, 2007). Such structures provide a variety of microenvironments, facilitating the co-existence of phylogenetically and physiologically diverse microorganisms (e.g., McCollom and Shock, 1997; Xia et al., 2011; Brazelton et al., 2012; Cao et al., 2014). In diffuser type chimneys, fluids percolate through horizontal channels in the chimney walls, resulting in hydrothermal fluid seepage from the entire chimney surface (Kormas et al., 2006). Overall, thermal and geochemical gradients are less pronounced, and the geochemical environment is more homogenous than in black smoker chimneys (Koski et al., 1994; Tivey, 2004, 2007).

Common to all hydrothermal chimneys is the formation of conditions favoring microbial growth (e.g., Jannasch and Mottl, 1985; Schrenk et al., 2008). In general, active vent sites are dominantly colonized by chemolithotrophic microorganisms (e.g., Harmsen et al., 1997; Brazelton et al., 2006; Zhou et al., 2009; Flores et al., 2011). From cultivation-based and metagenomic studies it is known that many vent-associated microorganisms are metabolically flexible – capable of utilizing more than one of the reduced chemical energy sources supplied by hydrothermal venting (e.g., Reysenbach, 2001; Eder and Huber, 2002; Campbell et al., 2006; Nakagawa and Takai, 2008; Dahle et al., 2013; Anderson et al., 2014; Mino et al., 2014; Perner et al., 2014; Meier et al., 2016, 2017). Temporal and spatial variations in venting intensity and fluid composition have been proposed to select for such metabolic flexibility (e.g., Huber et al., 2002; Perner et al., 2009, 2014; Amend et al., 2011; Dahle et al., 2013; Lin et al., 2016). The available, albeit scarce data on the *in situ* gene and protein expression of vent-associated microorganisms supports this hypothesis (e.g., Dahle et al., 2013; He et al., 2015; Stokke et al., 2015; Fortunato and Huber, 2016).

Within the scope of previous studies, we explored the diversity of microbial communities colonizing hydrothermal chimneys recovered from the Manus Basin back-arc spreading system. All analyzed samples were found to be colonized by various chemolithoautotrophic *Epsilonproteobacteria*, while the abundance and distribution of other putative primary producers (e.g., *Gammaproteobacteria, Zetaproteobacteria*, and *Aquificae*) was variable between structures (Reeves et al., 2014; Meier et al., 2017). Further, we could show that CO_2_ fixation via the reverse tricarboxylic acid (rTCA) cycle was the primary mode of carbon fixation on actively venting chimneys from the Manus Basin (Reeves et al., 2014). Here, we follow up on these studies using community proteogenomics to explore the genomic potential and *in situ* functional profiles of chimney colonizing communities. In order to gather direct insight on the effects of venting regime/fluid flow on microbial community composition and function, we analyzed two different hydrothermal chimney types sampled at the same vent site (Roman Ruins; RMR) and fed by a single fluid source (Reeves et al., 2011), but representing two different venting regimes due to different degrees of subsurface mixing of hydrothermal fluids and seawater: a diffuser (RMR-D) and a black smoker (RMR5).

## Materials and Methods

### Site Description and Sample Collection

We collected one diffusely venting chimney talus (RMR-D) and one black smoker spire (RMR5) found at 60 m distance from each other in the Roman Ruins vent field (Supplementary Figure S1; see also Reeves et al., 2014). The sampling site was located at ∼1700 m depth at the northeastern edge of the PACMANUS venting area of the Manus Basin back-arc spreading center of the Bismarck Sea, Papua New Guinea (Supplementary Figure S1). Samples were collected during expedition SO-216 on board the R/V *Sonne* (June–July 2011; Bach et al., 2011), with help of the remotely operated vehicle (ROV) *Quest 4000m* (MARUM, Bremen, Germany). Sample RMR5 corresponds to SO216-39ROV01 and sample RMR-D corresponds to SO216-53ROV03 (Bach et al., 2011). The black smoker spire (RMR5) was venting 324°C fluids prior to sampling. Reeves et al. (2014) report compositional data for the fluid venting from RMR5 as well as additional information on the geological setting and mineralogical composition of the two samples. During ROV ascent, samples were stored in closed bio-boxes to prevent contamination and mixing with sea-water. After retrieval, samples were preserved at -80°C until further analysis.

### DNA Extraction and Metagenome Sequencing

After samples were transferred to the home laboratory, large portions of chimney material (100–150 g) were collected from the outer 3–5 cm of chimney walls with a sterile spatula, and homogenized by stirring, mechanical crushing and vortexing prior to downstream treatments. DNA was extracted from 1.5 to 2.0 g of homogenized material according to a modified DNA extraction protocol published in Zhou et al. (1996). Briefly, 2.7 ml extraction buffer (see Zhou et al., 1996) and 5 μl Proteinase K (20 mg ml^-1^) were added per gram sample, and incubated for 30 min at 37°C on a rotary shaker. Thereafter, 0.6 ml 10% sodium dodecyl sulfate (SDS) per gram sample were added. After a 60 min incubation at 65°C on a rotary shaker, the extraction mixture was centrifuged for 10 min at 3220 × *g*. The supernatant was transferred to a new sample tube and mixed with an equal volume of chloroform:isoamyl alcohol. Centrifugation was repeated and the aqueous phase of the solution was transferred to another clean sample tube. The chloroform:isoamyl alcohol purification step was repeated twice. For DNA precipitation, 0.6 volumes isopropanol were added. The mixture was incubated at 22°C for 60 min before the DNA was pelleted by 45 min centrifugation at 3220 × *g*. The DNA pellet was washed twice with 10 ml cold, 70% molecular grade ethanol and finally re-suspended in 200 μl TE buffer. In total, 5.9 μg high-molecular weight DNA per g wet chimney material were extracted from RMR-D, while 1.5 μg g^-1^ were recovered from RMR5. At the Max Planck Genome Center (Cologne, Germany), DNA was fragmented by ultrasonication (Covaris S2 sonicator, Covaris, Woburn, MA, United States), followed by fragment size selection with SPRIselect beads (Beckman Coulter, United States) and metagenomic library preparation with the NEBNext Ultra DNA Library Prep Kit for Illumina (New England Biolabs, Ipswich, MA, United States) as instructed by the manufacturers. Paired-end (2 × 100 bp) metagenomes were sequenced on the Illumina HiSeq 2000 (RMR-D) and Illumina HiSeq 2500 (RMR5) sequencing platforms, respectively.

### Metagenome Analysis

Metagenome sequence reads were clipped to remove adaptor sequences and low quality bases (phred score 15, min. length 50 bp) using Nesoni^1^. Quality trimmed reads were uploaded to the MG-RAST platform (Meyer et al., 2008) for phylogenetic and functional whole-community classification. Phylogenetic classification was performed by BLAT-based comparison (Kent, 2002) against the NCBI RefSeq Database (Release 55). Functional classification was performed by BLAT-based search against the Clusters of Orthologous Groups (COG) database (Tatusov et al., 2000). For phylogenetic and functional classification, a maximum *e*-value of 1e^-5^, a minimum sequence identity of 60%, and a minimum alignment length of 15 amino acids (aa) was used.

*De novo* metagenomic assemblies were generated with the uneven depth assembler IDBA-UD (Peng et al., 2012). Minimal contig length was set to 1000 bp and *k-mers* were iterated between 21 and 91 in steps of 20. Descriptive statistics on metagenome sequence data and assemblies are provided in Supplementary Table S1. Metagenome assemblies were uploaded to the integrated microbial genomes and metagenomes (IMG/M) system for open reading frame (ORF) prediction and annotation. Phylogenetic classification of contigs was performed based on a BLAST search of predicted ORFs against the NCBI RefSeq Database, as described in Markowitz et al. (2012). Functional classification of predicted ORFs was performed by BLAST search against the COG database (Markowitz et al., 2012). ORFs of interest were identified via COG search, Pfam motive search (Finn et al., 2014) or by targeted protein BLAST searches (with BLASTP 2.2.26+, Altschul et al., 1997), and the annotation was manually curated. A phylogenetic tree of SoxY proteins was calculated based on metagenomic sequences obtained in this study together with the sequences used in Meier et al. (2017). The tree was calculated *de novo* based on a MAFFT alignment (v. 7.221, L-INS-I mode) of all SoxY protein sequences. Only positions conserved in at least 25% of the sequences were considered. Tree calculation was performed with FastTree (v. 2.1.9; Price et al., 2009) employing the Le-Gauscel model (-lg), using weighted joints (-bionj) and rescaling branches to optimize Gamma20 likelihood (-gamma). The obtained tree was visualized with ARB (Ludwig et al., 2004).

### Ribosomal RNA Gene Reconstruction

Small subunit (SSU) rRNA gene sequences were reconstructed from combined quality trimmed metagenomic reads of both libraries using PhyloFlash (v. 2.0^2^). Reads of each library were mapped onto the reconstructed sequences with a minimum identity of global alignment of 99% (using BBmap v. 36.02^3^) to estimate the relative abundance of each sequence in the respective dataset. Sequences with a coverage of less than one were regarded as not present in a sample and removed from further analysis. Shannon–Weaver and inverse Simpson diversity indices were calculated using the “diversity (species_count_per_site, index = “shannon”/“invsimpson”)” function, beta diversity was calculated with function “vegdist (species_count_per_site, binary = T)”, and Chao1 index with the function “estimateR (species_count_per_site)” in R-package “Vegan” (Oksanen et al., 2013).

### Nucleotide Accession Numbers

Metagenome assemblies are available under IMG Taxon IDs 3300003177 (RMR-D) and 3300003178 (RMR5), while the raw reads can be retrieved from ENA under the study accession number PRJEB24039.

### Protein Extraction

Protein extraction was performed from 7 g subsamples of homogenized chimney material according to a Tri Reagent (Sigma-Aldrich, Germany) and chloroform-based extraction protocol published in Stokke et al. (2012). Extracted protein was re-suspended in 40 μl of SDS-containing sample buffer (0.1 M Tris HCl pH 6.8, 10% SDS, 20% glycerol, 5% β-mercaptoethanol; see Teeling et al., 2012) and denaturated for 10 min at 90°C. After addition of 1 μl bromophenol blue, the denaturated protein extract was loaded on two separate lanes (i.e., technical duplicates) of a precast 4–20% gradient polyacrylamide SDS mini gel and separated at 150 V for 1 h. The gel was Coomassie Brilliant Blue (CBB)-stained, entire gel lanes were excised and cut into 10 equal pieces each, washed three times (200 mM NH_4_HCO_3_, 30% acetonitrile) and vacuum-dried before subsequent protein digestion with 2 μg ml^-1^ trypsin solution (Promega, United States) at 37°C overnight (Eymann et al., 2004).

### Mass Spectrometry and Data Analysis

After purification of peptide lysates with μ-C18 ZipTips (Millipore, United States), the peptide mix was separated by Nano HPLC (Easy-nLCII HPLC system, Thermo Fisher Scientific, Germany) and subjected to MS/MS analysis in an LTQ Orbitrap Velos mass spectrometer (Thermo Fisher Scientific, Germany) following a procedure adapted from Elsholz et al. (2012).

In brief, peptides were separated with self-packed analytical columns [C18-material (Luna 3u C18 100A; Phenomenex), 100 μm i.D. × 200 mm column] using a binary gradient from 1% buffer A [0.1% (vol/vol) acetic acid] to 75% buffer B [99.9% (vol/vol) acetonitrile, 0.1% (vol/vol) acetic acid] within 80 min and a flow rate of 300 nL min^-1^. The mass spectrometer was used in data-dependent MS/MS mode with a resolution of *R* = 30,000 for the full scan. MS/MS spectra were acquired for the 20 most intense precursor ions, and fragmented with collision-induced dissociation. The lockmass option was enabled during the runs.

MS/MS spectra were extracted and analyzed using the Sorcerer-Sequest software (Sorcerer v3.5, Sage-N Research; Sequest version v.27, rev. 11, Thermo-Finnigan, Thermo Fisher Scientific, Germany). A combined target-decoy database (total number of ORFs: 645,166) containing the amino acid sequences of all predicted protein-coding genes derived from the metagenomic information of both samples (322,541 coding sequences), 42 common laboratory contaminants, and reversed amino acid sequences of all protein-coding genes (322,583 decoys), was searched for protein identification. Search parameters were set as follows: peptide tolerance: 10 ppm; tolerance for fragment ions: 1 amu; band y-ion series; variable modification: oxidation of methionine, 15.99 Da, maximum three modifications per peptide. Evaluation of MS/MS-based peptide and protein identifications was performed with Scaffold version 4.0.4^4^. The following filter settings were applied: Parent mass tolerance: 10 ppm; Xcorr for doubly charged peptides 2.2, for triply charged peptides 3.3 and for quadruply charged peptides 3.75; Cn score 0.1. Using these settings, peptide false discovery rates (peptide FDRs, based on the number of decoy identifications) were below 0.1% throughout all experiments, and protein FDRs were 0.3–0.5%. Only proteins or protein groups that had at least two unique peptides assigned were considered to be identified.

For semi-quantitative analysis and comparison of relative protein abundances in both metagenome samples, normalized spectral abundance factors (NSAFs) were calculated (Zybailov et al., 2006) using total spectrum counts. For each protein or protein group, spectral count values were normalized to protein size (molecular weight in kDa) and to the sum of all spectral counts in the same sample (Supplementary File 1). The mass spectrometry proteomics data have been deposited to the ProteomeXchange Consortium via the PRIDE (Vizcaíno et al., 2016) partner repository with the dataset identifier PXD009105 and 10.6019/PXD009105.

## Results

### Phylogenetic Diversity of Chimney Colonizing Microbial Communities

**FIGURE 1:**
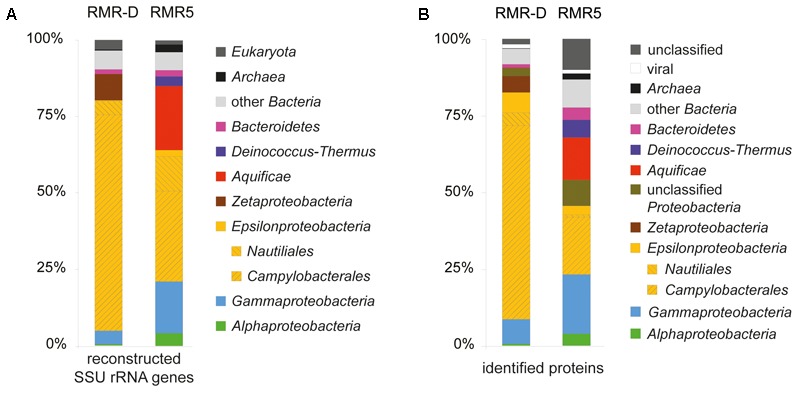
**(A)** Phylogenetic distribution of SSU rRNA gene OTUs reconstructed from the diffuser chimney (RMR-D) and black smoker chimney (RMR5) metagenomes. **(B)** Phylogenetic distribution of all proteins identified in the diffuser chimney (RMR-D) and black smoker chimney (RMR5) metaproteomes.

The composition of microbial communities colonizing the outer walls of the diffuser chimney talus (RMR-D) and the black smoker spire (RMR5) was evaluated by SSU rRNA gene reconstruction from metagenomic reads (**Figure 1A**). Consistent with results of previous studies (Reeves et al., 2014; Meier et al., 2017), both chimneys were colonized by *Epsilonproteobacteria*, mainly related to various genera within the *Campylobacterales*. Thermophiles related to the epsilonproteobacterial order *Nautiliales*, and the phyla *Aquificae* and *Deinococcus–Thermus*, as well as gammaproteobacterial and alphaproteobacterial sequences accounted for the majority of the remaining reconstructed SSU rRNA gene diversity in the RMR5 dataset (**Figure 1A**). *Zetaproteobacteria* were almost exclusively found on the diffuser RMR-D, while *Bacteroidetes*-related sequences were reconstructed from both chimney metagenomes (**Figure 1A**). Some reconstructed sequences were related to marine eukaryotes, mainly flagellated and ciliated protozoa. Related organisms have been previously detected in vent associated environments, but remain poorly characterized (Urich et al., 2014). *Archaea*, which were the focus of many studies investigating hydrothermal chimney colonization (e.g., Wang et al., 2009; Lincoln et al., 2013), including a chimney recovered from the Manus Basin (Takai et al., 2001), accounted for less than 2.5% of reconstructed SSU rRNA genes in either metagenome (**Figure 1A**). This observation is well in line with the low relative abundance of *Archaea* determined by catalyzed reporter deposition-fluorescence *in situ* hybridization (CARD-FISH) and intact polar lipid (IPL) analysis of surface crust samples previously obtained from these hydrothermal chimneys (Reeves et al., 2014).

**FIGURE 2:**
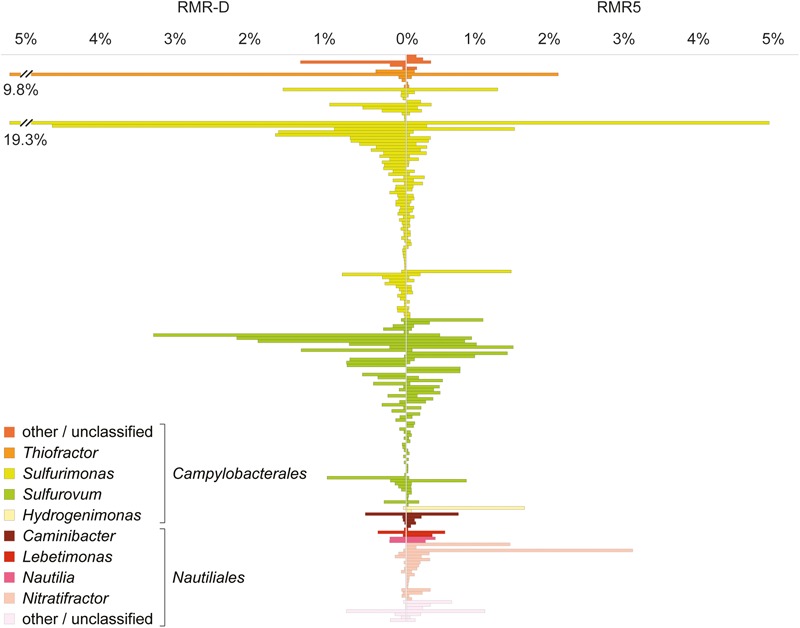
Abundance and phylogenetic classification of all epsilonproteobacterial SSU rRNA gene OTUs (*n* = 187) reconstructed from the diffuser chimney (RMR-D) and black smoker chimney (RMR5) metagenomes.

The phylogenetic classification of raw sequence reads and contigs assembled from the metagenomes (>1000 bp) mirrored the phylogenetic composition inferred from reconstructed SSU rRNA gene OTUs. Additionally, it revealed the presence of viral sequences in both metagenomes (Supplementary Figure S2). Likewise, the metaproteomic profile was phylogenetically consistent with the reconstructed SSU rRNA diversity: most proteins from RMR-D originated from *Campylobacterales*-related *Epsilonproteobacteria*, while epsilonproteobacterial, gammaproteobacterial and *Aquificae*-derived proteins were of similar relative abundance in the RMR-5 metaproteome (**Figure 1B**). Overall, the microbial community colonizing the black smoker chimney RMR5 was more diverse (Shannon–Weaver diversity index 4.9 for RMR5, vs. 3.9 for RMR-D) and had a considerably higher effective number of species than the community colonizing RMR-D (Inverse Simpson index 68.7 for RMR5, vs. 17 for RMR-D; Supplementary Table S2). However, an extensive overlap between microbial communities on RMR-D and RMR5 was detected (beta diversity of 0.37; Supplementary Table S2). 168 out of 367 reconstructed SSU rRNA gene OTUs were detected on both chimneys. These accounted for 88.9 and 47.5% of the total reconstructed SSU rRNA diversity on RMR-D and RMR5, respectively. The overlap was particularly pronounced within the *Epsilonproteobacteria*, with 119/187 reconstructed epsilonproteobacterial OTUs detected on both chimneys (**Figure 2**), accounting for 95.0% (RMR-D) and 81.9% (RMR5) of the total reconstructed epsilonproteobacterial diversity.

### The Metabolic Potential of Chimney Colonizing Microbial Communities

**FIGURE 3:**
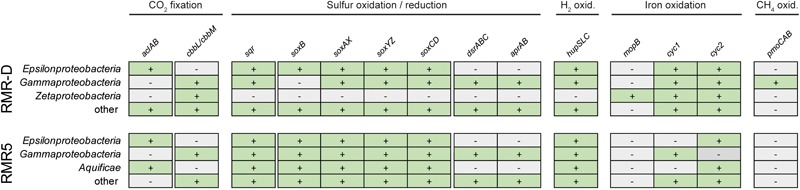
An overview of the genomic potential for carbon dioxide (CO_2_) fixation, sulfur compound oxidation and reduction, hydrogen (H_2_) oxidation, iron oxidation and methane (CH_4_) oxidation discovered by functional gene screening of the diffuser chimney (RMR-D) and black smoker chimney (RMR5) metagenomes. Presence of the respective marker genes in the designated phylogenetic group is indicated by a plus sign.

We mined the assembled metagenomic data for marker genes of different chemolithotrophic metabolisms and carbon fixation pathways to identify and classify putative functional guilds and primary producers within the chimney colonizing microbial communities. In the metagenome of the diffuser chimney RMR-D, marker genes for hydrogen oxidation (*hupSLC*), sulfide oxidation (*sqr*) and Sox-pathway dependent sulfur oxidation (*soxB, soxAX, soxYZ*, and *soxCD*) were found on epsilonproteobacterial and gammaproteobacterial contigs (**Figure 3**). Two operons encoding for the particulate methane monooxygenase (*pmoCAB*) were found on *Methylococcales-*related gammaproteobacterial contings from RMR-D (Supplementary File 1 and **Figure 3**). Various *Gammaproteobacteria* on RMR-D encoded genes for sulfur oxidation via the reverse dissimilatory sulfite reductase (rDSR) pathway (*dsrABC, aprAB*) (**Figure 3**). Furthermore, zetaproteobacterial molybdopterin oxidoreductases (*mopB*) and various proteobacterial cytochromes encoding genes putatively involved in neutrophilic Fe(II)-oxidation (*cyc1, cyc2*; Yarzábal et al., 2002) were identified in the RMR-D metagenome (**Figure 3**). In the metagenome recovered from chimney RMR5, the genetic potential for hydrogen (*hupSCL)*, sulfide (*sqr*) and sulfur compound oxidation via the SOX pathway (*soxB, soxAX, soxYZ*, and *soxCD*) was mainly found on contigs classified as *Epsilonproteobacteria, Gammaproteobacteria*, and *Aquificae*, while *Gammaproteobacteria* additionally encoded the potential for sulfur compound oxidation via the rDSR pathway (*dsrABC, aprAB*) (**Figure 3**). Genes indicative of an iron oxidation potential were detected less frequently in the RMR5 metagenome, and no indications for methane oxidizers were detected (**Figure 3** and Supplementary File 1). No evidence for the presence of methanogens, ammonia oxidizing archaea or bacteria, or nitrite oxidizing bacteria was detected on functional gene level in either metagenome.

The genomic potential for aerobic respiration was encoded by phylogenetically diverse microorganisms in both investigated metagenomes. We could identify genes encoding three types of terminal oxidases [cbb3-type (*ccoNOQP*), bd-type (*cydAB*), or aa3-type (*qoxABCD*) cytochrome *c* oxidases; Ferguson-Miller and Babcock, 1996] on a variety of bacterial and a few archaeal contigs (Supplementary File 1). Likewise, genes encoding enzymes involved in denitrification (Thöny-Meyer, 1997; Jones et al., 2008), including both the cytoplasmic (*narGHIJ*) and periplasmic (*napABC*) nitrate reductases, nitric oxide reductases (*norBC*) and nitrous oxide reductases (*nosZ*) were detected in both metagenomes, affiliating with members of various phylogenetic groups (Supplementary File 1). Furthermore, *dsrABC* and *aprAB* genes or operons phylogenetically affiliated to known sulfate or sulfur reducing microorganisms (*Deltaproteobacteria, Euryarchaeota*) were identified in the RMR-D metagenome (Supplementary File 1).

Marker genes for carbon fixation via the rTCA cycle (*aclAB*; Hügler and Sievert, 2011) were found on epsilonproteobacterial contigs in the RMR-D metagenome (**Figure 3** and Supplementary File 1), while marker genes for carbon fixation via the Calvin–Benson–Bassham (CBB) cycle (*ccbL*/*cbbM*; Hügler and Sievert, 2011) were found mainly on contigs affiliated with *Zetaproteobacteria* and *Gammaproteobacteria* (**Figure 3** and Supplementary File 1). In the RMR-5 metagenome, genes encoding for ribulose 1,5-bisphosphate carboxylase/oxygenase (RuBisCO; *cbbL*/*cbbM*) were found mainly on contigs of gammaproteobacterial origin (Supplementary File 1 and **Figure 3**), while the genomic potential for carbon fixation via the rTCA cycle was encoded by *Epsilonproteobacteria* and *Aquificae* (**Figure 3**). No genomic evidence for inorganic carbon fixation via the acetyl-CoA or Wood–Ljungdahl [WL] pathway, the 3-hydroxypropionate bicycle, the 3-hydroxypropionate/4-hydroxybutyrate cycle or the dicarboxylate/4-hydroxybutyrate cycle was found in either metagenome (Supplementary File 1).

### *In Situ* Metaproteomic Profiles of Hydrothermal Chimney Colonizing Microbial Communities

The *in situ* metabolic profile of chimney colonizing microbial communities was examined by a search of all recorded peptide spectra from both chimney metaproteomes against a database of all predicted ORFs. In total, we identified 2706 proteins. Thereof, 1802 were unique to RMR-D and 748 were unique to RMR5 (Supplementary Figure S4). 156 proteins were detected in both metaproteomes (Supplementary Figure S4), accounting for 27.7 and 15.5% of the identified metaproteomic profile in RMR-D and RMR5, respectively. Almost all highly abundant proteins from RMR-D were of epsilonproteobacterial origin and the majority had a predicted metabolic function (Supplementary Figure S3). The most highly abundant proteins from RMR5, on the other hand, were of diverse phylogenetic origin and predominantly lacked a functional prediction (Supplementary Figure S3). Overall, hypothetical proteins and conserved proteins of unknown function made up 41% of the proteomic profile from RMR5, while only 26% of the proteins identified from RMR-D had no assigned function.

**FIGURE 4:**
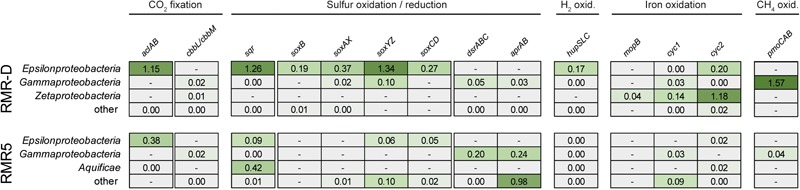
An overview of the *in situ* metaproteomic profile for carbon dioxide (CO_2_) fixation, sulfur compound oxidation and reduction, hydrogen (H_2_) oxidation, iron oxidation and methane (CH_4_) oxidation in the diffuser chimney (RMR-D), and black smoker chimney (RMR5) metaproteomes. The number indicates the cumulative normalized spectral abundance factors (NSAFs) for the respective gene group in the corresponding phylogenetic group. The data is underlined by a heatmap, for easier identification of relative protein abundances. A darker shade of green indicates a higher relative abundance of the corresponding protein clusters. Absence of detected proteins in the metaproteomes for any given protein cluster in the corresponding phylogenetic group is represented by a minus in a gray box.

On both chimneys, the abundant genomic potential for hydrogen oxidation was poorly reflected in the metaproteome (**Figure 4**). Only five proteins corresponding to the 160 genes from the *hupSLC* operons were detected, all of them of epsilonproteobacterial origin (Supplementary File 1). On RMR-D, *Zetaproteobacteria* seem to have been involved in iron oxidation, as indicated by the identification of molybdopterin oxidoreductases (MopB), as well as Cyc1 and Cyc2-type cytochromes in the metaproteomes (**Figure 4**). Additionally, proteins corresponding to one of the two detected *Methylococcales*-related particulate methane monooxygenase gene clusters were highly abundant in the metaproteome, suggesting methane oxidation to occur in RMR-D (Supplementary Figure S3 and **Figure 4**). Overall, proteins related to sulfur oxidation (the SQR, and proteins of the SOX and rDSR pathways) constituted the functional group with the highest relative abundance in the metaproteomes of both chimneys (**Figure 4**). Of the few identified *dsrABC* and *aprAB* genes predicted to originate from sulfate or sulfur reducing microorganisms (*Deltaproteobacteria* and *Euryarchaeota*), only one euryarchaeal AprA was detected in the metaproteomes (**Figure 4** and Supplementary File 1).

Among the three types of terminal oxidases encoded in both metagenomes (cbb3-type, bd-type, and aa3-type cytochrome *c* oxidases), the high-affinity cbb3-type oxidases showed the highest relative protein abundance in metaproteomes from both chimneys, while no low-affinity aa3-type oxidases were detected (Supplementary Figure S5). All bd-type cytochrome *c* oxidases detected in the metaproteomes were of epsilonproteobacterial origin, while the cbb3-type cytochrome *c* oxidases showed a more diverse phylogenetic affiliation (Supplementary File 1). The relative abundance of proteins involved in denitrification was overall lower than the relative abundance of oxygen-reducing terminal oxidases (Supplementary File 1). The majority of detected denitrification-related proteins originated from epsilonproteobacterial contigs (Supplementary File 1).

Primary production via the CBB pathway seems to have been of little importance on either chimney, as ATP-citrate lyase which served as marker for the rTCA pathway showed a much higher relative abundance in metaproteomes from both chimneys than ribulose-1,5-bisphosphate carboxylase/oxygenase (RuBisCO), the key enzyme of the CBB cycle (**Figure 4**). In total, only three RuBisCO large subunit proteins (of gamma- and zetaproteobacterial origin) were among the identified proteins and all were of low relative abundance (Supplementary File 1 and **Figure 4**). These observations support the dominance of rTCA cycle-mediated primary production on these hydrothermal chimneys, previously hypothesized based on δ^13^C values of microbial IPL side chains (Reeves et al., 2014).

### The Effect of Venting Regime on Epsilonproteobacterial *In Situ* Protein Expression Profiles

Since the epsilonproteobacterial community colonizing RMR-D and RMR5 was largely overlapping (**Figure 2**), we compared the relative abundance profile of all identified epsilonproteobacterial proteins between RMR-D and RMR5 to assess possible effects of venting regime on their *in situ* function. In total, 1253 proteins accounting for 74.3% of the metaproteomic profile on RMR-D could be assigned to epsilonproteobacterial contigs, while this was the case for 231 proteins, accounting for 22.1% of the metaproteomic profile, from RMR5. Notably, 133 of the 156 proteins identified in both metaproteomes were of epsilonproteobacterial origin. Thus, epsilonproteobacterial proteins essentially accounted for the entire shared metaproteomic profile between RMR-D and RMR5.

**FIGURE 5:**
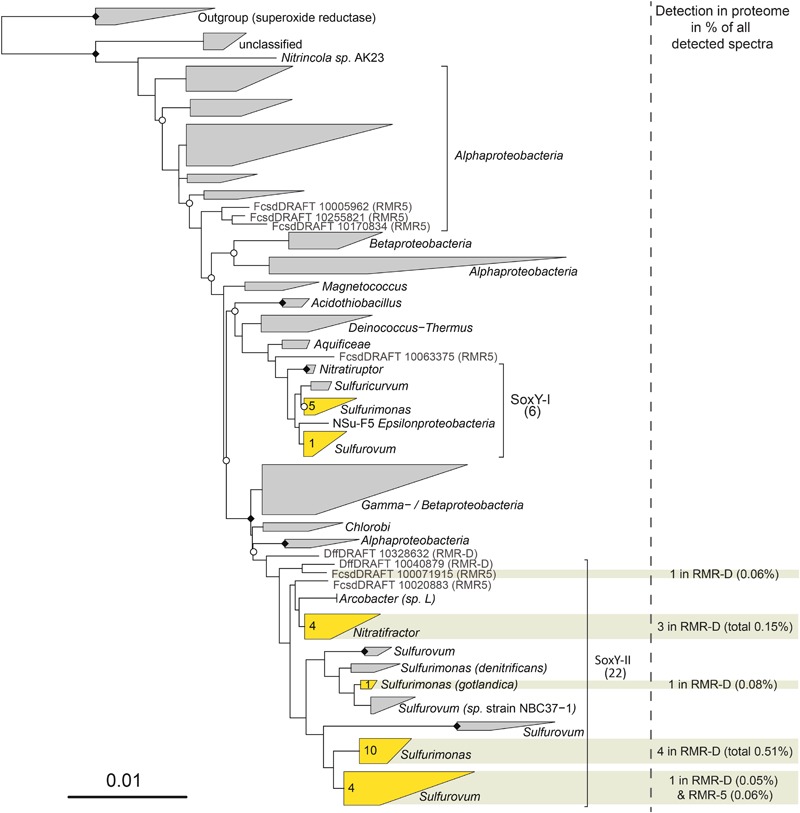
Phylogenetic tree of SoxY protein sequences, based on a SoxY guide tree from Meier et al. (2017). Clusters containing sequences obtained in this study are depicted in yellow, with the number of sequences given in the cluster. Individual sequences obtained in this study are depicted in gray. Clusters with proteins detected in the metaproteomes are highlighted in ocher, and the cumulative normalized spectral abundance factors (NSAFs) for the SoxY proteins, together with the total number of detected proteins and their origin is given on the right hand side.

A COG function could be assigned to 78.5 and 91.7% of the epsilonproteobacterial proteins identified on RMR-D and RMR5, respectively (Supplementary Figure S6). On both chimneys, epsilonproteobacterial proteins involved in energy production and conversion constituted the functional category with the highest relative abundance (Supplementary Figure S6). Among those, the high diversity of the identified sulfur anion binding proteins (SoxY) on RMR-D stood out: in total, 10 epsilonproteobacterial SoxY proteins were identified in the metaproteomes. *Epsilonproteobacteria* encode two distinct SoxY copies: a conserved copy (SoxY-I), encoded in the *soxABXYZ* operon, and a more diverse copy (SoxY-II) from the *soxYZCD* operon (Meier et al., 2017). In the metaproteomes, only SoxY proteins from the more diverse cluster (SoxY-II) were detected (**Figure 5**). Furthermore, in both epsilonproteobacterial metaproteomes, proteins related to cell motility, ribosomal structure, protein translation, posttranslational modification and protein turnover accounted for the majority of the remaining metaproteomic profile (Supplementary Figure S6). Notably, cell motility related proteins (mainly associated with flagellar structure and biosynthesis) accounted for a larger fraction of the epsilonproteobacterial metaproteomic profile on the black smoker chimney RMR5.

## Discussion

We used community proteogenomics to investigate the composition and *in situ* activity of microorganisms colonizing two hydrothermal vent chimneys from the Roman Ruins vent field in the Manus Basin back-arc spreading center. Hydrothermal chimneys is this area vent fluids characterized by low pH, low H_2_ and CH_4_, and high H_2_S concentrations (Reeves et al., 2011, 2014). However, different venting regimes (diffusive vs. focused fluid flow) create different geochemical and physical environments, which in turn resulted in the colonization of the outer chimney walls by distinct microbial communities (**Figure 1**).

Common to both investigated chimneys is the pivotal role of sulfur-oxidation driven chemosynthesis, which is a typical trait of most volcanic-hosted hydrothermal systems (e.g., Amend et al., 2011; Dahle et al., 2013; Cao et al., 2014; Lin et al., 2016), whereas life at vents strongly influenced by serpentinization is supported primarily by H_2_ and CH_4_ (e.g., Amend et al., 2011; Brazelton et al., 2012). Also alike for RMR-D and RMR5 is the absence of evidence for methanogenic activity, and the overall low contribution of anaerobic metabolisms (denitrification, sulfate reduction) to either communities’ functional profile. The available H_2_ concentrations, which are very low in hydrothermal fluids venting from Roman Ruins and other vent fields in the Manus Basin (Reeves et al., 2011), might have been too low to support methanogenesis, even if anaerobic niches were present in the analyzed chimney surface biofilms. A similar scenario was suggested by Flores et al. (2011) with regard to the absence of methanogens in chimneys recovered at the Lucky Strike hydrothermal vent field. However, we cannot exclude that methanogenesis, sulfate or sulfur reduction or other anaerobic metabolisms do occur in deeper chimney layers, which were not sampled for the purpose of this study. The high-affinity cbb3-type cytochrome *c* oxidases were the respiratory complexes with the highest relative abundance in the metaproteomes of both chimney colonizing microbial communities analyzed in this study, suggesting predominantly microaerobic conditions. Similar observations have been made in previous studies of hydrothermal chimneys and sediments (Xia et al., 2011; Dahle et al., 2013; Urich et al., 2014). In diffuse fluids, on the other hand, the aa3-type cytochrome *c* oxidases were reported to be the most highly expressed terminal oxidases (Fortunato and Huber, 2016). Thus, oxygen availability is likely an important discriminating factor between solid surface and aqueous hydrothermal environments with comparable temperatures and reductant concentrations.

The high microbial diversity and more even relative abundance distribution of microbes colonizing RMR5 is in line with the thermal and geochemical stratification within black smoker chimney walls (e.g., Tivey, 2004). The dominant colonizers we identified on RMR5 are related to chemolithoautotrophs among the *Epsilonproteobacteria, Gammaproteobacteria*, and *Aquificales*. In addition, colonization by thermophiles, e.g., members of the *Aquificales* and *Deinococcus–Thermus*, was detected both in the metagenomic and metaproteomic dataset from RMR5. This colonization pattern directly supports the first order control of temperature on microbial colonization of black smoker chimneys suggested by Schrenk et al. (2008), and is in line with microbial community profiles of black smoker chimneys reported in previous studies (e.g., Xia et al., 2011; Cao et al., 2014; Ferrera et al., 2014). The microbial community colonizing the diffuser chimney RMR-D, in contrast, was of comparatively low complexity and dominated by a few *Sulfurimonas*- and *Sulfurovum*-related epsilonproteobacterial phylotypes (**Figure 2**). Overall, this colonization pattern phylogenetically resembles *Epsilonproteobacteria*-dominated biofilms recovered from other hydrothermal chimneys and sediments venting sulfide rich fluids (Dahle et al., 2013; Urich et al., 2014; Stokke et al., 2015).

Overall, the *in situ* metaproteomic profile of both chimney colonizing microbial communities correlates well with the composition of hydrothermal fluids at the sampling site. The discrepancies we observed between the genomic potential and the *in situ* metaproteomic profile, however, are unexpectedly large. For example, the numerous RuBisCO and hydrogenase encoding genes detected in both metagenomes was in stark contrast to the marginal abundance of the corresponding proteins in the metaproteomes. It has previously been shown that an absolute quantity of about 100 fmol is necessary for successful protein detection (Silva et al., 2006). Considering the high (micro)diversity of the chimney colonizing microbial communities (**Figure 2**), alongside the fact that we were able to identify only between ∼6–12% of all recorded peptide spectra (Supplementary Table S1), it is likely that many proteins were present at absolute quantities that were below our detection threshold. The higher abundance of identified spectra in the RMR-D metaproteome, which originates from a less diverse and less even microbial community is in line with this hypothesis. Additionally, the detection of membrane bound proteins, like the here described epsilonproteobacterial hydrogenases (Sievert et al., 2008), is challenging and the applied protein isolation method has to be considered inherently biased against such proteins. Moreover, it is possible that the metagenomic data does not represent only the active community and intact cells, since extracellular DNA can persist in the environment long after cell death. For example, Carini et al. (2017) have shown that on average 40% of the DNA recovered from soil samples can be attributed to relic DNA, rather than the active microbial community. While these methodological issues certainly can have affected our metagenomic and metaproteomic data, the discrepancy between the metabolic potential and the identified functional profile in our dataset remains larger than discrepancies in a comparable study of hydrothermal chimney colonizing microbial communities at Loki’s Castle (Dahle et al., 2013; Stokke et al., 2015). At Loki’s Castle, a simultaneous high expression of epsilonproteobacterial genes involved in sulfur and hydrogen oxidation was detected. At Roman Ruins, in contrast, sulfur-oxidation was clearly the dominant energy source for the colonizing *Epsilonproteobacteria*. The different functional profiles of these phylogenetically similar communities are most likely linked to the differences in the geochemical composition of the venting hydrothermal fluids, namely the low concentrations of molecular hydrogen in fluids venting from the Roman Ruins vent field (Reeves et al., 2011, 2014). Based on our results, we propose that the interpretation of frequently applied DNA-based functional gene profiling approaches has to be approached with more caution (e.g., Xia et al., 2011), as no direct link could be drawn between the genetic potential and the *in situ* function of the metabolically versatile vent-associated microbiome in our study. The metabolic flexibility of vent-dwelling microorganisms is likely maintained in response to the frequent fluctuations in hydrothermal fluid composition selecting for functionally diverse microorganisms, but not all detected metabolic pathways are simultaneously active.

The clear dominance of sulfur-oxidation related proteins over proteins related to other oxidative chemolithotrophic metabolisms, like hydrogen and iron oxidation (**Figure 4**), suggests that niche partitioning on RMR5 was not primarily based on differential substrate utilization. Instead, various phylogenetically distinct colonizers on RMR5 seem to have been functionally redundant. The overall high phylogenetic diversity in RMR5 thus rather reflects the availability of multiple microniches differentiated by, e.g., temperature and pH, and is not directly linked to a higher functional diversity. On RMR-D, substrate-based niche differentiation was clearly present between sulfur-oxidizing *Epsilonproteobacteria* and iron-oxidizing *Zetaproteobacteria*, with the less abundant, but more diverse gammaproteobacterial community contributing to both of these processes, and in addition performing aerobic methane oxidation (**Figure 4**).

Interestingly, the chimney colonizing *Gammaproteobacteria* and *Alphaproteobacteria* hardly seem to have been utilizing their metabolic potential for inorganic carbon fixation. The majority of the here detected gammaproteobacterial community was phylogenetically related to various members of the order *Chromatiales* (Supplementary File 1), for which genomic data as well as isolated representatives are scarce. However, recently it has been recognized that many sulfur- and/or hydrogen-oxidizing representatives of this large and diverse order can grow chemoheterotrophically or mixotrophically (e.g., Flood et al., 2015; Mußmann et al., 2017). The same is true for the here dominant *Rhizobiales*- and *Rhodobacterales*-related *Alphaproteobacteria* (Supplementary File 1; e.g., Sorokin et al., 2005). Together with the *Bacteroidetes*, for which the polysaccharide-utilization related protein SusD was identified in both metaproteomes (Supplementary File 1), they were likely responsible for the utilization and recycling of organic carbon compounds supplied by the epsilonproteobacterial, zetaproteobacterial and *Aquificales*-related primary producers.

Generally, diffuse venting regimes supply more chemical energy per unit of time and chimney wall volume, since the fluid flow is percolative, rather than diffusion controlled (Kormas et al., 2006). We thus postulate that the diffuse venting regime provided a sufficient flux of reduced iron species and methane to support the development of an iron- and methane-oxidizing microbial community on RMR-D, while this was not the case for the focused venting regime in RMR5. Furthermore, the homogeneous, high energy supply in RMR-D seems to have selected for the dominance of few well adapted phenotypes.

Interestingly, despite the apparent functional coherency of the epsilonproteobacterial community on RMR-D, a relatively high level of (micro)diversity was still maintained (**Figure 2**). In our previous study of diffuse hydrothermal fluids from the Manus Basin (Meier et al., 2017), we suggested that one of the major drivers for diversification within the epsilonproteobacterial community is their adaptation to different concentrations of hydrogen sulfide or the availability of other sulfur compounds, which is reflected in the diversification of the SoxY protein, involved in sulfur-species transformations occurring via the Sox-pathway (Friedrich et al., 2000). While SoxY has already been identified as the most highly expressed Sox-pathway protein in other studies (Urich et al., 2014; Stokke et al., 2015), lacking functional data, we were previously not able to confirm that the diversified copy of SoxY (SoxY-II) is truly expressed. The metaproteomes obtained in this study revealed that indeed only the diversified version of epsilonproteobacterial SoxY protein (SoxY-II) was detectable, confirming its functionality. Furthermore, the simultaneous detection of multiple different SoxY-II proteins simultaneously in the diffuse venting RMR-D chimney (**Figure 5**) supports our hypothesis on the role of SoxY in energy niche adaptation of *Sulfurimonas* and *Sulfurovum* species by utilizing either different sulfur substrates or different concentrations of the same substrate (Meier et al., 2017).

## Author Contributions

PP, AM, and WB collected samples. PP and DM performed the analysis of metagenomic data with the help of MR and HG-V. SM produced and analyzed the proteomic data with the help of CH, DB, and TS. PP, RA, and AM conceived and wrote the manuscript with the help of all authors.

## Conflict of Interest Statement

The authors declare that the research was conducted in the absence of any commercial or financial relationships that could be construed as a potential conflict of interest.

## Footnotes

1 http://bioinformatics.net.au/software.nesoni.shtml

2 https://github.com/HRGV/phyloFlash

3 https://sourceforge.net/projects/bbmap/

4 http://www.proteomesoftware.com

## References

[B1] Altschul S. F. Madden T. L. Schaffer A. A. Zhang J. Zhang Z. Miller W. (1997). Gapped BLAST and PSI-BLAST: a new generation of protein database search programs. *Nucleic Acids Res.* 25 3389–3402. 9254694 10.1093/nar/25.17.3389 PMC146917

[B2] Amend J. P. McCollom T. M. Hentscher M. Bach W. (2011). Metabolic energy for chemolithoautotrophs in peridotite-hosted and basalt-hosted hydrothermal systems. *Geochim. Cosmochim. Acta* 75 5736–5748.

[B3] Anderson R. E. Sogin M. L. Baross J. A. (2014). Evolutionary strategies of viruses, bacteria and archaea in hydrothermal vent ecosystems revealed through metagenomics. *PLoS One* 9:e109696. 10.1371/journal.pone.0109696 25279954 PMC4184897

[B4] Bach W. Dubilier N. Borowski C. Breuer C. Brunner B. Franke P. (2011). *Report and Preliminary Results of RV SONNE Cruise SO-216 Townsville (Australia)–Makassar (Indonesia), June 14–July 23, 2011. BAMBUS, Back-Arc Manus Basin Underwater Solfataras. Universität Bremen*. Available at: http://elib.suub.uni-bremen.de/edocs/00102250-1.pdf

[B5] Brazelton W. J. Nelson B. Schrenk M. O. (2012). Metagenomic evidence for H_2_ oxidation and H_2_ production by serpentinite-hosted subsurface microbial communities. *Front. Microbiol.* 2:268 10.3389/fmicb.2011.00268 PMC3252642 22232619

[B6] Brazelton W. J. Schrenk M. O. Kelley D. S. Baross J. A. (2006). Methane-and sulfur-metabolizing microbial communities dominate the Lost City hydrothermal field ecosystem. *Appl. Environ. Microbiol.* 72 6257–6270. 16957253 10.1128/AEM.00574-06 PMC1563643

[B7] Campbell B. J. Engel A. S. Porter M. L. Takai K. (2006). The versatile 𝜀-proteobacteria: key players in sulphidic habitats. *Nat. Rev. Microbiol.* 4 458–468. 16652138 10.1038/nrmicro1414

[B8] Cao H. Wang Y. Lee O. O. Zeng X. Shao Z. Qian P. Y. (2014). Microbial sulfur cycle in two hydrothermal chimneys on the Southwest Indian ridge. *mBio* 5:e00980-13. 10.1128/mBio.00980-13 24473131 PMC3903282

[B9] Carini P. Marsden P. J. Leff J. W. Morgan E. E. Strickland M. S. Fierer N. (2017). Relic DNA is abundant in soil and obscures estimates of soil microbial diversity. *Nat. Microbiol.* 2:16242. 10.1038/nmicrobiol.2016.242 27991881

[B10] Dahle H. Roalkvam I. Thorseth I. H. Pedersen R. B. Steen I. H. (2013). The versatile *in situ* gene expression of an *Epsilonproteobacteria*-dominated biofilm from a hydrothermal chimney. *Environ. Microbiol. Rep.* 5 282–290. 10.1111/1758-2229.12016 23584970

[B11] Eder W. Huber R. (2002). New isolates and physiological properties of the *Aquificales* and description of *Thermocrinis albus* sp. nov. *Extremophiles* 6 309–318. 12215816 10.1007/s00792-001-0259-y

[B12] Elsholz A. K. Turgay K. Michalik S. Hessling B. Gronau K. Oertel D. (2012). Global impact of protein arginine phosphorylation on the physiology of *Bacillus subtilis*. *Proc. Natl. Acad. Sci. U.S.A.* 109 7451–7456. 10.1073/pnas.1117483109 22517742 PMC3358850

[B13] Eymann C. Dreisbach A. Albrecht D. Bernhardt J. Becher D. Gentner S. (2004). A comprehensive proteome map of growing *Bacillus subtilis* cells. *Proteomics* 4 2849–2876. 15378759 10.1002/pmic.200400907

[B14] Ferguson-Miller S. Babcock G. T. (1996). Heme/copper terminal oxidases. *Chem. Rev.* 96 2889–2908. 11848844 10.1021/cr950051s

[B15] Ferrera I. Banta A. B. Reysenbach A.-L. (2014). Spatial patterns of *Aquificales* in deep-sea vents along the Eastern Lau Spreading Center (SW Pacific). *Syst. Appl. Microbiol.* 37 442–448. 10.1016/j.syapm.2014.04.002 24862554

[B16] Finn R. D. Bateman A. Clements J. Coggill P. Eberhardt R. Y. Eddy S. R. (2014). The Pfam protein families database. *Nucleic Acids Res.* 42 D222–D230. 24288371 10.1093/nar/gkt1223 PMC3965110

[B17] Flood B. E. Jones D. S. Bailey J. V. (2015). *Sedimenticola thiotaurini* sp. nov., a sulfur-oxidizing bacterium isolated from salt marsh sediments, and emended descriptions of the genus *Sedimenticola* and *Sedimenticola selenatireducens*. *Int. J. Syst. Evol. Microbiol.* 65 2522–2530. 10.1099/ijs.0.000295 25944805

[B18] Flores G. E. Campbell J. H. Kirshtein J. D. Meneghin J. Podar M. Steinberg J. I. (2011). Microbial community structure of hydrothermal deposits from geochemically different vent fields along the Mid-Atlantic Ridge. *Environ. Microbiol.* 13 2158–2171. 10.1111/j.1462-2920.2011.02463.x 21418499

[B19] Fortunato C. S. Huber J. (2016). Coupled RNA-SIP and metatranscriptomics of active chemolithoautotrophic communities at a deep-sea hydrothermal vent. *ISME J.* 10 1925–1938. 10.1038/ismej.2015.258 26872039 PMC5029171

[B20] Friedrich C. G. Quentmeier A. Bardischewsky F. Rother D. Kraft R. Kostka S. (2000). Novel genes coding for lithotrophic sulfur oxidation of *Paracoccus pantotrophus* GB17. *J. Bacteriol.* 182 4677–4687. 10940005 10.1128/jb.182.17.4677-4687.2000 PMC111341

[B21] Harmsen H. Prieur D. Jeanthon C. (1997). Distribution of microorganisms in deep-sea hydrothermal vent chimneys investigated by whole-cell hybridization and enrichment culture of thermophilic subpopulations. *Appl. Environ. Microbiol.* 63 2876–2883. 16535655 10.1128/aem.63.7.2876-2883.1997 PMC1389210

[B22] He Y. Feng X. Fang J. Zhang Y. Xiao X. (2015). Metagenome and metatranscriptome revealed a highly active and intensive sulfur cycle in an oil-immersed hydrothermal chimney in Guaymas Basin. *Front. Microbiol.* 6:1236. 10.3389/fmicb.2015.01236 26617579 PMC4639633

[B23] Huber J. A. Butterfield D. A. Baross J. A. (2002). Temporal changes in archaeal diversity and chemistry in a mid-ocean ridge subseafloor habitat. *Appl. Environ. Microbiol.* 68 1585–1594. 11916672 10.1128/AEM.68.4.1585-1594.2002 PMC123862

[B24] Hügler M. Sievert S. M. (2011). Beyond the Calvin cycle: autotrophic carbon fixation in the ocean. *Annu. Rev. Mar. Sci.* 3 261–289. 10.1146/annurev-marine-120709-142712 21329206

[B25] Jannasch H. W. Mottl M. J. (1985). Geomicrobiology of deep-sea hydrothermal vents. *Science* 229 717–725. 17841485 10.1126/science.229.4715.717

[B26] Jones C. M. Stres B. Rosenquist M. Hallin S. (2008). Phylogenetic analysis of nitrite, nitric oxide, and nitrous oxide respiratory enzymes reveal a complex evolutionary history for denitrification. *Mol. Biol. Evol.* 25 1955–1966. 10.1093/molbev/msn146 18614527

[B27] Kent W. J. (2002). BLAT–the BLAST-like alignment tool. *Genome Res.* 12 656–664. 11932250 10.1101/gr.229202 PMC187518

[B28] Kormas K. A. Tivey M. K. Von Damm K. Teske A. (2006). Bacterial and archaeal phylotypes associated with distinct mineralogical layers of a white smoker spire from a deep-sea hydrothermal vent site (9° N, East Pacific Rise). *Environ. Microbiol.* 8 909–920. 16623747 10.1111/j.1462-2920.2005.00978.x

[B29] Koski R. A. Jonasson I. R. Kadko D. C. Smith V. K. Wong F. L. (1994). Compositions, growth mechanisms, and temporal relations of hydrothermal sulfide-sulfate-silica chimneys at the northern Cleft segment, Juan de Fuca Ridge. *J. Geophys. Res.* 99 4813–4832.

[B30] Lin T. J. Ver Eecke H. C. Breves E. A. Dyar M. D. Jamieson J. W. Hannington M. D. (2016). Linkages between mineralogy, fluid chemistry, and microbial communities within hydrothermal chimneys from the Endeavour Segment, Juan de Fuca Ridge. *Geochem. Geophys. Geosyst.* 17 300–323. 10.1002/2015GC006091 PMC6094386 30123099

[B31] Lincoln S. A. Bradley A. S. Newman S. A. Summons R. E. (2013). Archaeal and bacterial glycerol dialkyl glycerol tetraether lipids in chimneys of the Lost City Hydrothermal Field. *Org. Geochem.* 60 45–53.

[B32] Ludwig W. Strunk O. Westram R. Richter L. Meier H. Yadhukumar (2004). ARB: a software environment for sequence data. *Nucleic Acids Res.* 32 1363–1371. 14985472 10.1093/nar/gkh293 PMC390282

[B33] Markowitz V. M. Chen I. M. A. Chu K. Szeto E. Palaniappan K. Grechkin Y. (2012). IMG/M: the integrated metagenome data management and comparative analysis system. *Nucleic Acids Res.* 40 D123–D129. 10.1093/nar/gkr975 22086953 PMC3245048

[B34] McCollom T. M. Shock E. L. (1997). Geochemical constraints on chemolithoautotrophic metabolism by microorganisms in seafloor hydrothermal systems. *Geochim. Cosmochim. Acta* 61 4375–4391. 11541662 10.1016/s0016-7037(97)00241-x

[B35] Meier D. V. Bach W. Girguis P. R. Gruber-Vodicka H. R. Reeves E. P. Richter M. (2016). Heterotrophic *Proteobacteria* in the vicinity of diffuse hydrothermal venting. *Environ. Microbiol.* 8 4348–4368. 10.1111/1462-2920.13304 27001712

[B36] Meier D. V. Pjevac P. Bach W. Hourdez S. Girguis P. R. Vidoudez C. (2017). Niche partitioning of diverse sulfur-oxidizing bacteria at hydrothermal vents. *ISME J.* 11 1545–1558. 10.1038/ismej.2017.37 28375213 PMC5520155

[B37] Meyer F. Paarmann D. D’Souza M. Olson R. Glass E. M. Kubal M. (2008). The metagenomics RAST server–a public resource for the automatic phylogenetic and functional analysis of metagenomes. *BMC Bioinformatics* 9:386. 10.1186/1471-2105-9-386 18803844 PMC2563014

[B38] Mino S. Kudo H. Takayuki A. Sawabe T. Takai K. Nakagawa S. (2014). *Sulfurovum aggregans* sp. nov., a novel hydrogen-oxidizing, thiosulfate-reducing chemolithoautotroph within the *Epsilonproteobacteria* isolated from a deep-sea hydrothermal vent chimney at the Central Indian Ridge, and an emended description of the genus *Sulfurovum*. *Int. J. Syst. Evol. Microbiol.* 64 3195–3201. 10.1099/ijs.0.065094-0 24966202

[B39] Mußmann M. Pjevac P. Krüger K. Dyksma S. (2017). Genomic repertoire of the *Woeseiaceae*/*JTB255*, cosmopolitan and abundant core members of microbial communities in marine sediments. *ISME J.* 11 1276–1281. 10.1038/ismej.2016.185 28060363 PMC5437919

[B40] Nakagawa S. Takai K. (2008). Deep-sea vent chemoautotrophs: diversity, biochemistry and ecological significance. *FEMS Microbiol. Ecol.* 65 1–14. 10.1111/j.1574-6941.2008.00502.x 18503548

[B41] Oksanen J. Blanchet F. G. Kindt R. Legendre P. Michin P. R. O’Hara R. B. (2013). *vegan: Community Ecology Package. R Package Version 2.0-10*. Available at: http://CRAN.R-project.org/package=vegan

[B42] Peng Y. Leung H. C. Yiu S. M. Chin F. Y. (2012). IDBA-UD: a de novo assembler for single-cell and metagenomic sequencing data with highly uneven depth. *Bioinformatics* 28 1420–1428. 10.1093/bioinformatics/bts174 22495754

[B43] Perner M. Bach W. Hentscher M. Koschinsky A. Garbe-Schönberg D. Streit W. R. (2009). Short-term microbial and physico-chemical variability in low-temperature hydrothermal fluids near 5°S on the Mid-Atlantic Ridge. *Environ. Microbiol.* 11 2526–2541. 10.1111/j.1462-2920.2009.01978.x 19558512

[B44] Perner M. Gonnella G. Kurtz S. LaRoche J. (2014). Handling temperature bursts reaching 464°C: different microbial strategies in the Sisters Peak hydrothermal chimney. *Appl. Environ. Microbiol.* 80 4585–4598. 24837379 10.1128/AEM.01460-14 PMC4148794

[B45] Price M. N. Dehal P. S. Arkin A. P. (2009). FastTree: computing large minimum evolution trees with profiles instead of a distance matrix. *Mol. Biol. Evol.* 26 1641–1650. 10.1093/molbev/msp077 19377059 PMC2693737

[B46] Reeves E. P. Seewald J. S. Saccocia P. Bach W. Craddock P. R. Shanks W. C. (2011). Geochemistry of hydrothermal fluids from the PACMANUS, Northeast Pual and Vienna Woods hydrothermal fields, Manus Basin, Papua New Guinea. *Geochim. Cosmochim. Acta* 75 1088–1123.

[B47] Reeves E. P. Yoshinaga M. Y. Pjevac P. Goldenstein N. Peplies J. Meyerdierks A. (2014). Microbial lipids reveal carbon assimilation patterns on hydrothermal sulfide chimneys. *Environ. Microbiol.* 16 3515–3532. 10.1111/1462-2920.12525 24905086

[B48] Reysenbach A.-L. (2001). “Phylum BI. Aquificae *phy. nov*,” in *Bergey’s Manual of Systematic Bacteriology* eds Garrity G. M. Boone D. R. Castenholz R. W. (New York, NY: Springer) 359–367.

[B49] Rickard D. Knott R. Duckworth R. Murton B. (1994). Organ pipes, beehive diffusers and chimneys at the Broken Spur hydrothermal sulphide deposits, 29°N MAR. *Minerol. Mag.* 58A 774–775.

[B50] Schrenk M. O. Holden J. F. Baross J. A. (2008). “Magma to-microbe networks in the context of sulfide hosted microbial ecosystems,” in *The Subseafloor Biosphere at Mid-Ocean Ridges* eds Wilcock W. S. D. DeLong E. F. Kelley D. S. Baross J. A. Cary S. C. (Washington, DC: American Geophysical Union) 233–258.

[B51] Sievert S. M. Scott K. M. Klotz M. G. Chain P. S. G. Hauser L. J. Hemp J. (2008). Genome of the epsilonproteobacterial chemolithoautotroph *Sulfurimonas denitrificans*. *Appl. Environ. Microbiol.* 74 1145–1156. 18065616 10.1128/AEM.01844-07 PMC2258580

[B52] Silva J. C. Gorenstein M. V. Li G.-Z. Vissers J. P. C. Geromanos S. J. (2006). Absolute quantification of proteins by LCMSE a virtue of parallel ms acquisition. *Mol. Cell. Proteomics* 5 144–156. 16219938 10.1074/mcp.M500230-MCP200

[B53] Sorokin D. Y. Tourova T. P. Muyzer G. (2005). *Citreicella thiooxidans* gen. nov., sp. nov., a novel lithoheterotrophic sulfur-oxidizing bacterium from the Black Sea. *Syst. Appl. Microbiol.* 28 679–687. 16261857 10.1016/j.syapm.2005.05.006

[B54] Stokke R. Dahle H. Roalkvam I. Wissuwa J. Daae F. L. Tooming-Klunderud A. (2015). Functional interactions among filamentous *Epsilonproteobacteria* and *Bacteroidetes* in a deep-sea hydrothermal vent biofilm. *Environ. Microbiol.* 17 4063–4077. 10.1111/1462-2920.12970 26147346

[B55] Stokke R. Roalkvam I. Lanzen A. Haflidason H. Steen I. H. (2012). Integrated metagenomic and metaproteomic analyses of an ANME-1-dominated community in marine cold seep sediments. *Environ. Microbiol.* 14 1333–1346. 10.1111/j.1462-2920.2012.02716.x 22404914

[B56] Takai K. Komatsu T. Inagaki F. Horikoshi K. (2001). Distribution of archaea in a black smoker chimney structure. *Appl. Environ. Microbiol.* 67 3618–3629. 11472939 10.1128/AEM.67.8.3618-3629.2001 PMC93063

[B57] Tatusov R. L. Galperin M. Y. Natale D. A. Koonin E. V. (2000). The COG database: a tool for genome-scale analysis of protein functions and evolution. *Nucleic Acids Res.* 28 33–36. 10592175 10.1093/nar/28.1.33 PMC102395

[B58] Teeling H. Fuchs B. M. Becher D. Klockow C. Gardebrecht A. Bennke C. M. (2012). Substrate-controlled succession of marine bacterioplankton populations induced by a phytoplankton bloom. *Science* 336 608–611. 10.1126/science.1218344 22556258

[B59] Thöny-Meyer L. (1997). Biogenesis of respiratory cytochromes in bacteria. *Microbiol. Mol. Biol. Rev.* 61 337–376. 9293186 10.1128/mmbr.61.3.337-376.1997 PMC232615

[B60] Tivey M. K. (2004). “Environmental conditions within active seafloor vent structures: sensitivity to vent fluid composition and fluid flow,” in *The Subseafloor Biosphere at Mid-Ocean Ridges* eds Wilcock W. S. D. DeLong E. F. Kelley D. S. Baross J. A. Cary S. C. (Washington, DC: American Geophysical Union) 137–152.

[B61] Tivey M. K. (2007). Generation of seafloor hydrothermal vent fluids and associated mineral deposits. *Oceanography* 20 50–65.

[B62] Urich T. Lanzén A. Stokke R. Pedersen R. B. Bayer C. Thorseth I. H. (2014). Microbial community structure and functioning in marine sediments associated with diffuse hydrothermal venting assessed by integrated meta-omics. *Environ. Microbiol.* 16 2699–2710. 10.1111/1462-2920.12283 24112684

[B63] Vizcaíno J. A. Csordas A. del-Toro N. Dianes J. A. Griss J. Lavidas I. (2016). 2016 update of the PRIDE database and related tools. *Nucleic Acids Res.* 44 D447–D456. 10.1093/nar/gkv1145 26527722 PMC4702828

[B64] Wang S. Xiao X. Jiang L. Peng X. Zhou H. Meng J. (2009). Diversity and abundance of ammonia-oxidizing archaea in hydrothermal vent chimneys of the Juan de Fuca Ridge. *Appl. Environ. Microbiol.* 75 4216–4220. 10.1128/AEM.01761-08 19395559 PMC2698363

[B65] Xia W. Wang F. Guo L. Chen Z. Sievert S. M. Meng J. (2011). Comparative metagenomics of microbial communities inhabiting deep-sea hydrothermal vent chimneys with contrasting chemistries. *ISME J.* 5 414–426. 10.1038/ismej.2010.144 20927138 PMC3105715

[B66] Yarzábal A. Brasseur G. Ratouchniak J. Lund K. Lemesle-Meunier D. DeMoss J. A. (2002). The high-molecular-weight cytochrome *c cyc2* of *Acidithiobacillus ferrooxidans* is an outer membrane protein. *J. Bacteriol.* 184 313–317. 11741873 10.1128/JB.184.1.313-317.2002 PMC134758

[B67] Zhou H. Li J. Peng X. Meng J. Wang F. Ai Y. (2009). Microbial diversity of a sulfide black smoker in main endeavour hydrothermal vent field, Juan de Fuca Ridge. *J. Microbiol.* 47 235–247. 10.1007/s12275-008-0311-z 19557339

[B68] Zhou J. Bruns M. A. Tiedje J. M. (1996). DNA recovery from soils of diverse composition. *Appl. Environ. Microbiol.* 62 316–322. 8593035 10.1128/aem.62.2.316-322.1996 PMC167800

[B69] Zybailov B. Mosley A. L. Sardiu M. E. Coleman M. K. Florens L. Washburn M. P. (2006). Statistical analysis of membrane proteome expression changes in *Saccharomyces cerevisiae*. *J. Proteome Res.* 5 2339–2347. 16944946 10.1021/pr060161n

