# Supplementary figures and images for: Metaproteogenomic Profiling of Microbial Communities Colonizing Actively Venting Hydrothermal Chimneys
Source: Front Microbiol. 2018 Apr 6;9:680. doi: 10.3389/fmicb.2018.00680 (PMC5904459; doi:10.3389/fmicb.2018.00680)

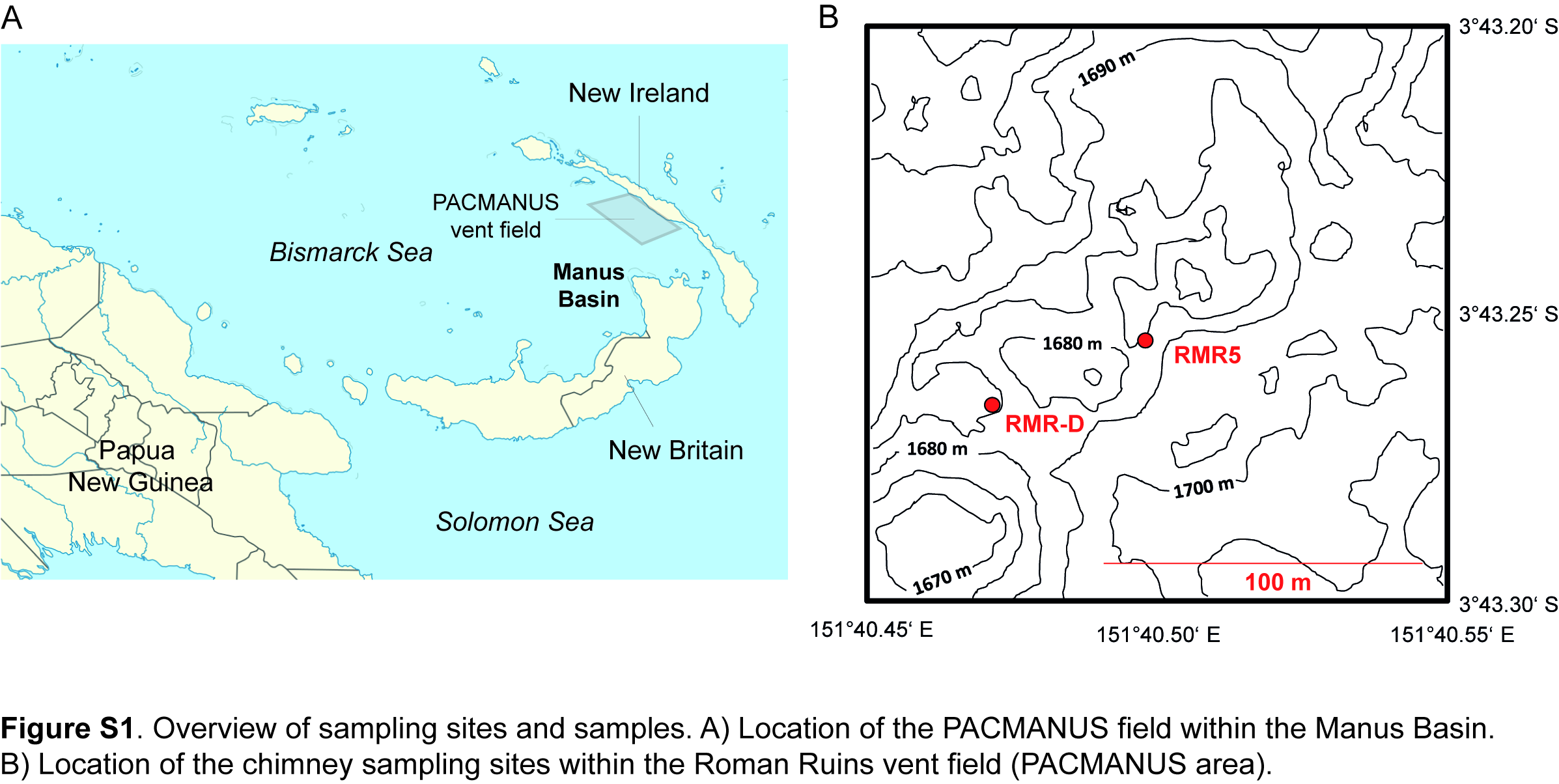

Supplement: Supplementary file 2 [file Image_1.tif]

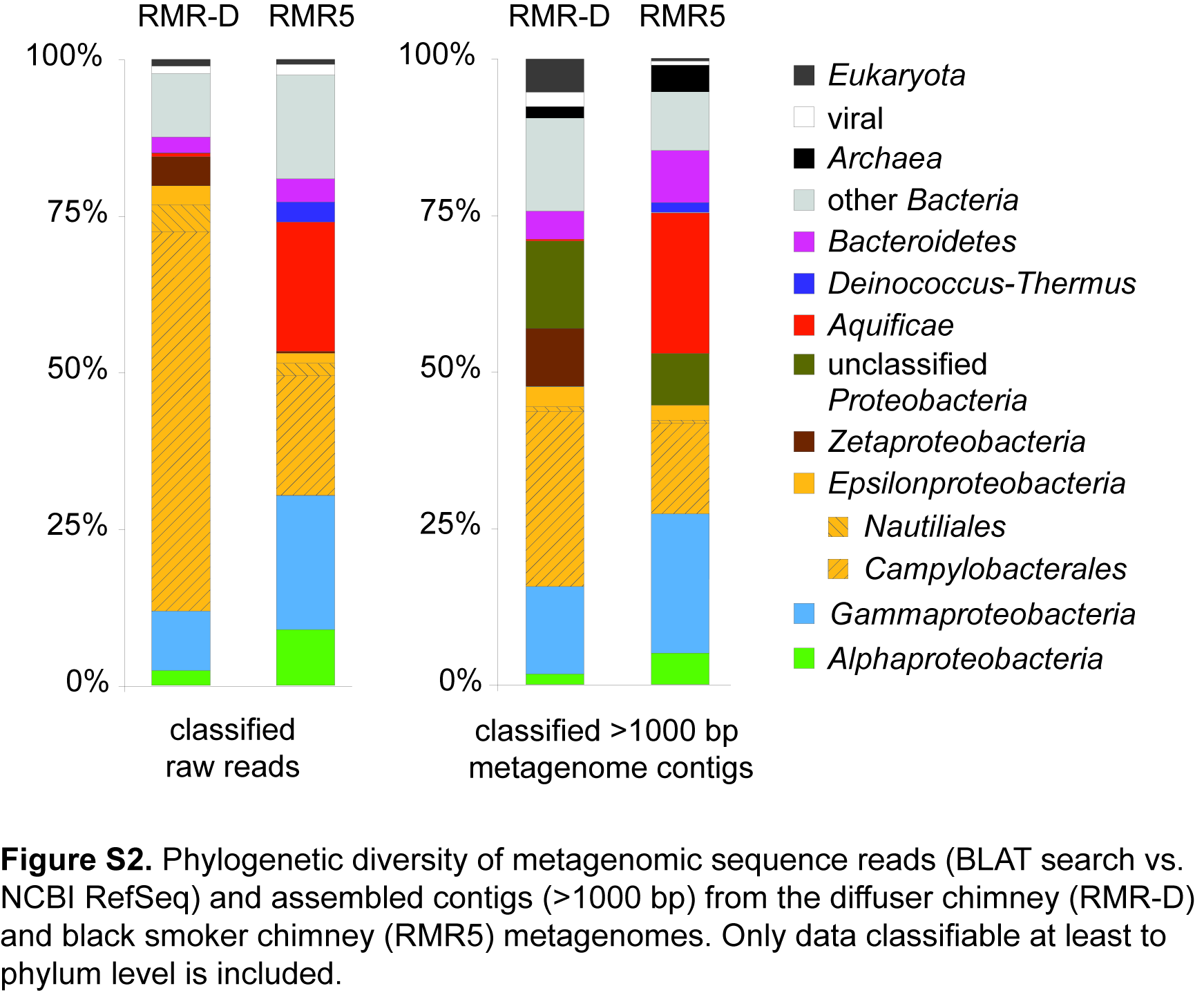

Supplement: Supplementary file 3 [file Image_2.tif]

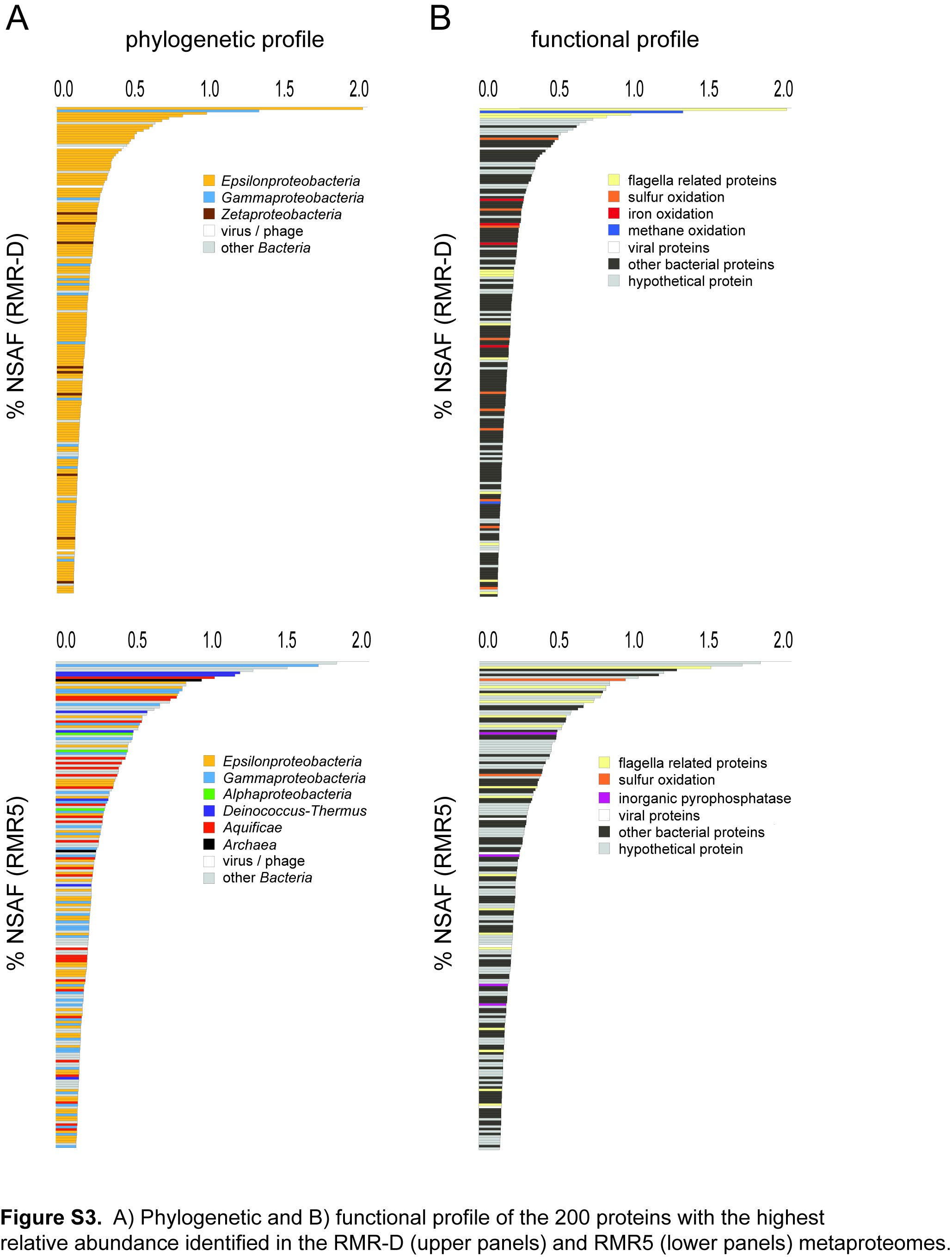

Supplement: Supplementary file 4 [file Image_3.tif]

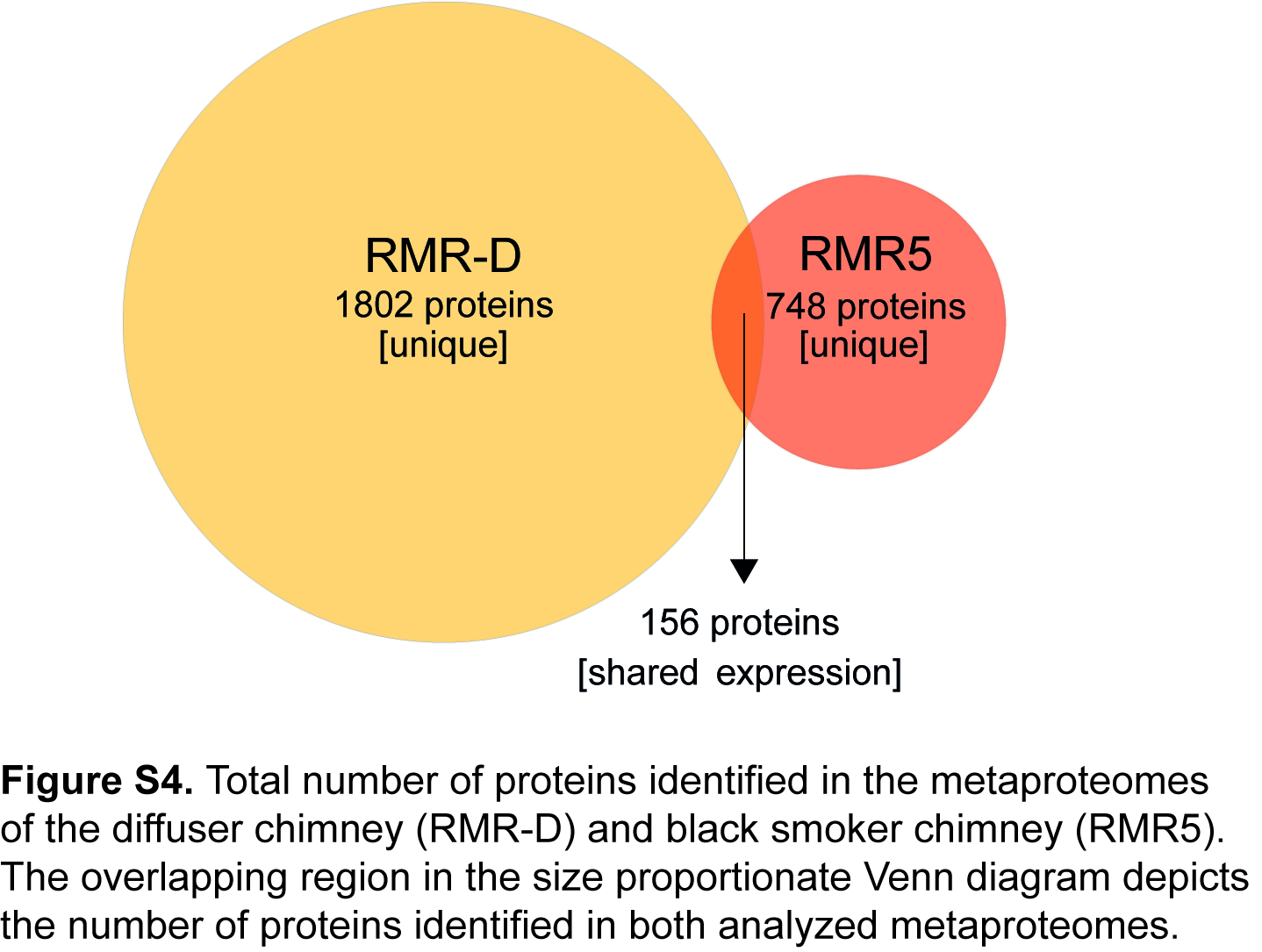

Supplement: Supplementary file 5 [file Image_4.tif]

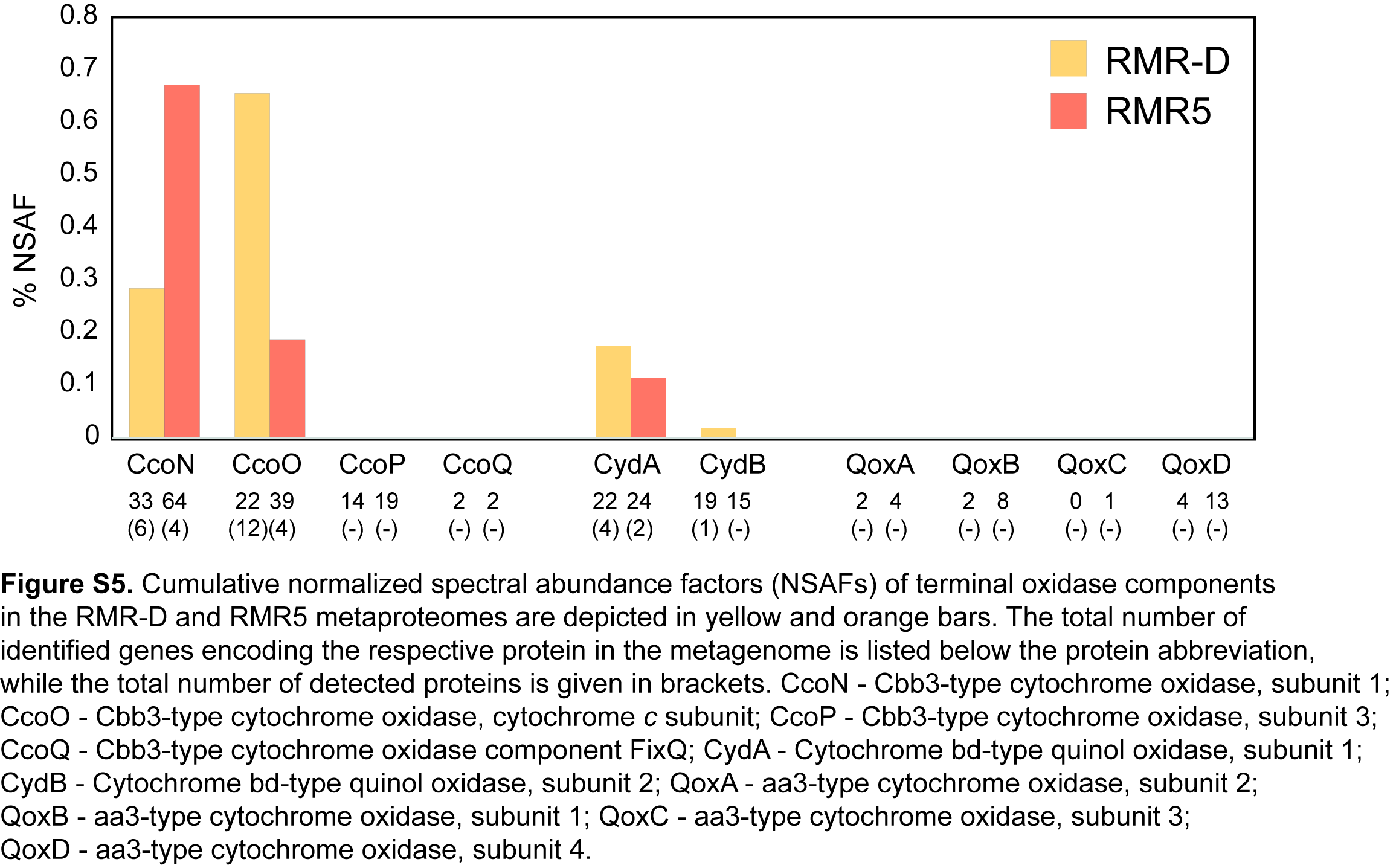

Supplement: Supplementary file 6 [file Image_5.tif]

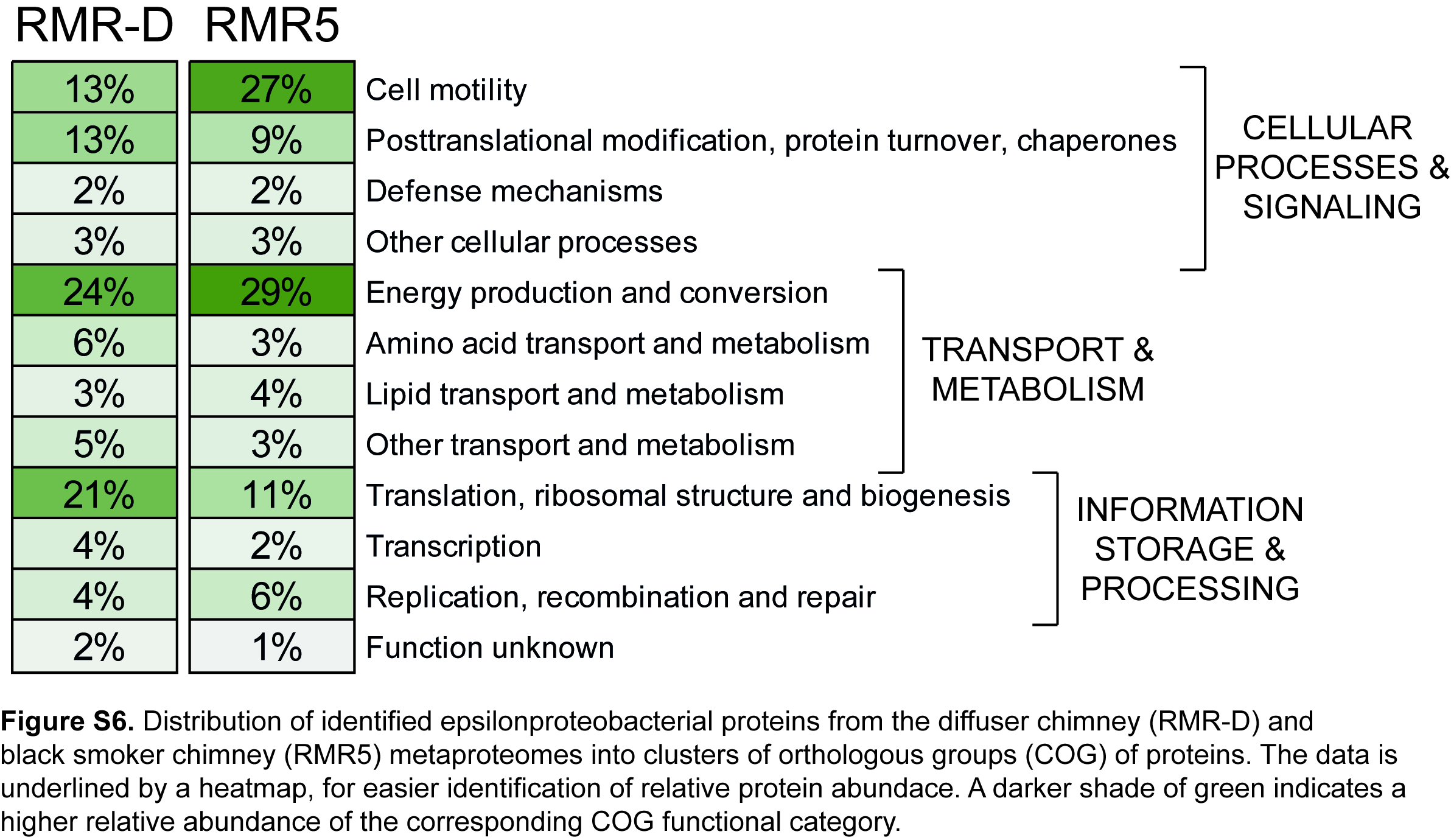

Supplement: Supplementary file 7 [file Image_6.tif]
